# Biosynthesis of Rishirilide B

**DOI:** 10.3390/antibiotics7010020

**Published:** 2018-03-07

**Authors:** Philipp Schwarzer, Julia Wunsch-Palasis, Andreas Bechthold, Thomas Paululat

**Affiliations:** 1 Pharmaceutical Biology and Biotechnology, University of Freiburg, Stefan-Meier-Str. 19, 79104 Freiburg im Breisgau, Germany; philipp.schwarzer@pharmazie.uni-freiburg.de (P.S.); Wujul@gmx.de (J.W.-P.); 2 Organic Chemistry, University of Siegen, Adolf-Reichwein-Str. 2, 57068 Siegen, Germany

**Keywords:** streptomyces, rishirilide, biosynthesis, polyketides

## Abstract

Rishirilide B was isolated from *Streptomyces rishiriensis* and *Streptomyces bottropensis* on the basis of its inhibitory activity towards alpha-2-macroglobulin. The biosynthesis of rishirilide B was investigated by feeding experiments with different ^13^C labelled precursors using the heterologous host *Streptomyces albus* J1074::cos4 containing a cosmid encoding of the gene cluster responsible for rishirilide B production. NMR spectroscopic analysis of labelled compounds demonstrate that the tricyclic backbone of rishirilide B is a polyketide synthesized from nine acetate units. One of the acetate units is decarboxylated to give a methyl group. The origin of the starter unit was determined to be isobutyrate.

## 1. Introduction

Polyketides represent a large and diverse group of natural products from different biological sources such as bacteria, plants and fungi. Numerous polyketides are pharmacologically useful and in clinical use in many areas of application [1].

**Figure 1:**
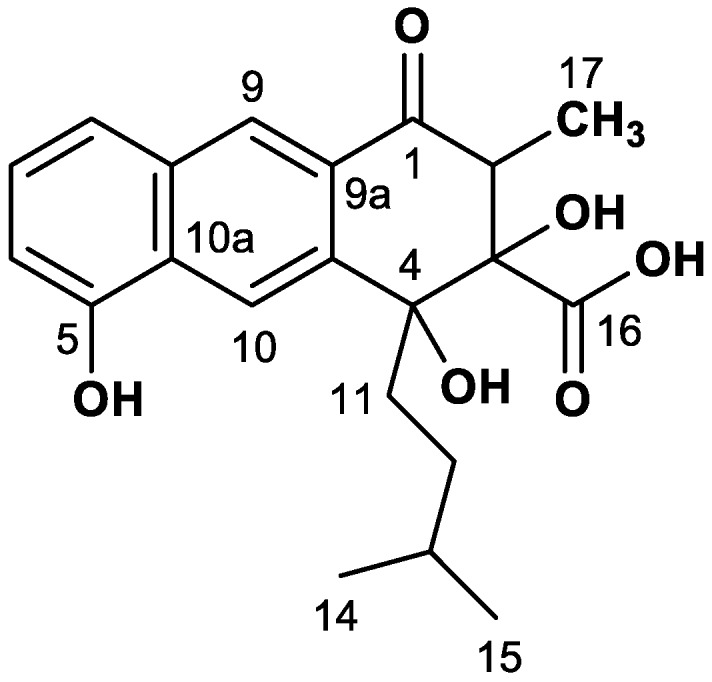
Structure of rishirilide B.

Rishirilide B (Figure 1), a product of a type II polyketide synthase (PKS), was first isolated from *Streptomyces rishiriensis* OFR-1056 in 1984 [2]. Later, *Streptomyces bottropensis* (formerly *Streptomyces* sp. Gö C4/4) was described as another producer of rishirilide B [3]. Rishirilide B inhibits alpha-2-macroglobulin, a plasma protein that effects the blood coagulation system by inhibiting a large variety of proteinases. Thus alpha-2-macroglobulin inhibition is an effective mechanism to prevent and treat fibrinolytic accentuated thrombosis [3]. Structurally, rishirilide B is a tricyclic compound with an isopentyl sidechain, and further substitutions including one methyl, one carboxlic acid, and three hydroxyl groups. The isopentyl side chain is uncommon for aromatic polyketides. This branched chain may originate from activated isobutyrate and could be the starter unit of polyketide synthesis [4]. The origin of this starter unit might be valine-derived isobutyl-CoA as described in literature for other natural compounds, such as tautomycin and virginiamycin M [5,6]. Isobutyryl-CoA derives from l-valine via transamination and decarboxylation [7].

Aromatic polyketide formation undergoes a complex building process under the control of the PKS. CoA activated carboxylic acids are assembled via decarboxylative Claisen condensation by an iteratively used set of proteins leading to the formation of a highly reactive polyketide chain. Within this process, many variables determine the unique order of events and therewith the final structure of the emerging polyketide. The variables include the choice of starter units, the determination of chain length as well as cyclisation patterns. Post PKS events, induced by tailoring enzymes, increase structural variation [4,8]. A review by Staunton and Weissman provides a complete overview about polyketides [9]. The gene cluster responsible for Rishirilide B production contains 28 ORF’s that encode a type II PKS, tailoring enzymes, regulatory proteins, and transporters [3].

Insights in the early formation process of polyketides can be obtained by incorporation studies with labelled precursors. These experiments lead to substantial information about the starter unit and the number of incorporated extender units and also indicate the order of events during polyketide formation and give valuable hints about possible rearrangement steps that might occur throughout the biosynthesis. In this way, the carbon backbone of many known natural products like malonomicin, polyketomycin and clavulanic acid could be examined [10,11,12]. Recent incorporation studies with labelled [1-^13^C]acetate, [2-^13^C]acetate, and [1,2-^13^C_2_]acetate and [^13^c_6_]-l-isoleucine on trixocarcin, a natural compound produced by *Streptomyces bottropensis* DO-45 led to the identification of eight extender units and to 2-methylbutyryl-CoA, a previously unknown starter unit which derives from isoleucine [13]. Here we describe incorporation studies with [1-^13^C]acetate, [2-^13^C]acetate, and [1,2-^13^C_2_]acetate, as well as [^13^c_5_,^15^n_1_]-l-valine that clarify the origin of all carbon atoms in rishirilide B. These experiments also give insight into the rearrangement process that occur during rishirilide biosynthesis.

## 2. Results

**Figure 2:**
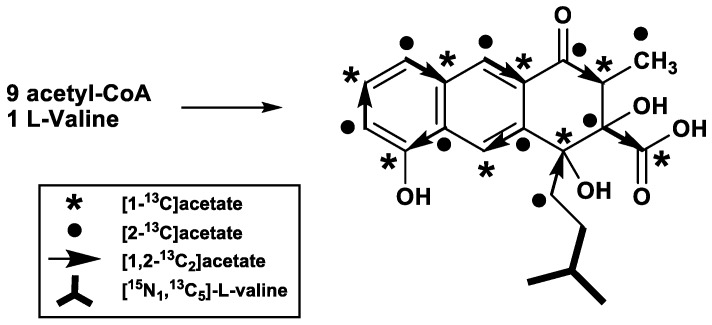
Labelling positions from feeding experiments using different labelled acetates and l-valine.

A cosmid encoding the entire rishirilide B gene cluster was transformed into *Streptomyces albus* J1074, yielding *S. albus* J1074::cos4. Stable heterologous production of rishirilide B was observed with *S. albus* J1074::cos4 and therefore all feeding experiments have been carried out with this strain. The origin of each carbon atom in rishirilide B was determined by supplementing the production medium with [1-^13^C]-, [1,2-^13^C_2_]-, [2-^13^C]acetate, and [^13^c_5_,^15^n_1_]-l-valine (Figure 2 and Table 1, assignment in Table S1).

Incorporation studies with [1-^13^C]acetate show ^13^C isotopic labelling in positions C-2, C-4, C-5, C-7, C-8a, C-9a, C-10 and C-16 as detected from ^13^C NMR signal enhancements (Table S2, Figures S1 and S2). Feeding of uniformly labelled [1,2-^13^C_2_]acetate reveals eight intact acetate units (Table S3, Figures S3–S5). When denoted according to the direction of the proposed biosynthetic pathway, these acetate units correspond to C11/C4, C4a/C-10, C-10a/C-5, C-6/C-7, C-8/C-8a, C-9/C-9a, C-1/C-2 and C-3/C-16. C-17 was proposed to come from methionine, but a feeding experiment with l-[methyl-^13^c]methionine did not produce a labelled position in rishirilide B (Table S4, Figure S6). After feeding of [2-^13^C]acetate, enrichment in positions C-1, C-3, C-4a, C-6, C-8, C-9, C-10a, C-11 and C-17 was observed (Table S5, Figures S7 and S8). This implies that an acetate unit affords the C-17 methyl group after decarboxylation. This result also demonstrates that a nonaketide is the precursor to the tricyclic backbone of rishirilide B.

The C-12–C-15 moiety, which represents the starter unit of the polyketide, was not affected by any feeding experiment with labelled acetate or methionine, indicating that it derives from a different precursor. However, feeding experiments with [^13^c_5_,^15^n_1_]-l-valine led to a mass increase of +4 (*m*/*z*) compared to unlabeled rishirilide B (Figure S9). ^13^C-NMR spectroscopic analysis likewise showed enrichments at positions C-12, C-13, C-14, and C-15 (Table S6, Figures S10–S13). Both MS and NMR data are consistent with a starter unit that is a valine derived isobutyryl-CoA.

## 3. Discussion

The biosynthetic origin of all carbon atoms of rishirilide B has been determined through labelled precursor feeding experiments. The tricyclic skeleton shows the expected isotopic labelling pattern that arises from a nonaketide that adopts an S-mode folding prior to condensation and aromatization [14]. Decarboxylation of one acetate shortens the polyketide, resulting in a residual methyl group. The starter unit arises from isobutyryl-CoA, which in turn is derived from valine. Interestingly, when feeding with [^13^c_5_,^15^n]-l-valine was performed, we observed strong enrichment of C12-C15 but also weak enrichment pattern in other carbons similar to [1,2-^13^C_2_]acetate incorporation. Despite feeding the labelled precursors in several pulses, some l-valine degraded to acetate.

**Figure 3:**
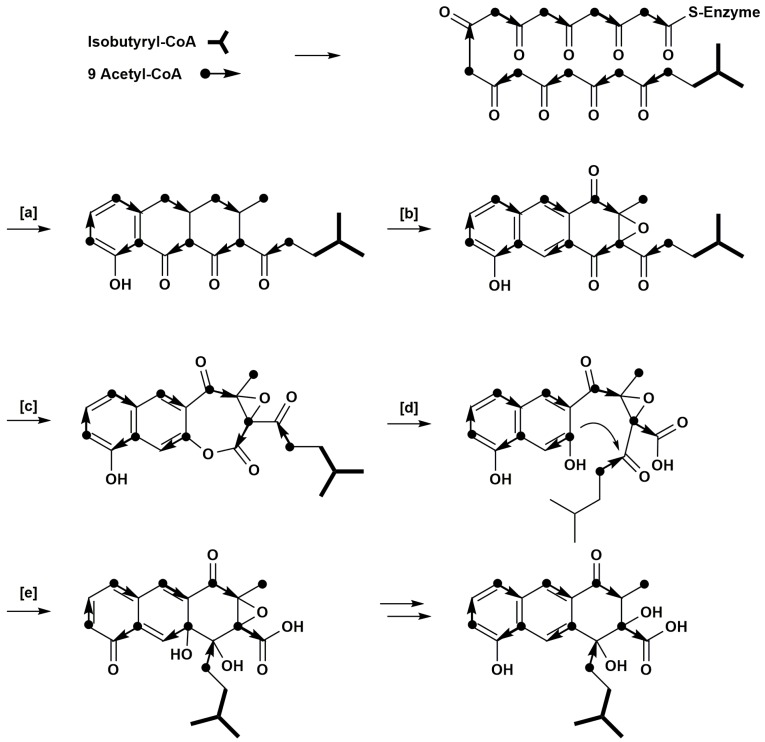
Proposed pathway of rishirilide B biosynthesis. (**a**) cyclisation and decarboxylation; (**b**) aromatization, oxidation; (**c**) Bayer-Villiger oxidation; (**d**) hydrolytic ring opening; (**e**) aldole condensation.

The labelling pattern arising from acetate feeding indicates that a rearrangement occurs during rishirilide B biosynthesis, otherwise C-17 and C-3/C-16 could not show the detected isotopic enrichments. We propose that a Bayer-Villiger type oxidation followed by hydrolytic ring opening and an aldol condensation would produce the labelling pattern detected in our feeding experiments (Figure 3). A similar sequence of Bayer-Villiger oxidation and hydrolysis is proposed for the conversion of questin to desmethylsulochrin [15]. Likewise, Bayer-Villiger oxidations play a role in the biosynthesis of premithramycin B-lactone or murayaquinone, respectively [16,17]. Enterocin, produced by *Streptomyces maritimus*, also has an unprecedented carbon skeleton that is derived from an aromatic polyketide biosynthetic pathway. Its caged tricyclic, nonaromatic core is derived from a linear poly-beta-ketide precursor that undergoes a Favorskii-like oxidative rearrangement [18,19].

Further examples on molecular level show that Bayer-Villiger rearrangements can be catalyzed by oxygenases, like the monooxygenases GilOII in Gilovarcin biosynthesis and BexE in BE-7585A biosynthesis [20,21]. Several enzymes like the luciferase like monooxygenase RslO1 and the putative monooxygenase of the antibiotic biosynthesis monooxygenase (ABM) superfamily RslO4, are potential candidates involved in the rearrangement. An experimental confirmation is still pending.

## 4. Materials and Methods 

### 4.1. Bacterial Strains and Cultivation

Experiments were performed in *Streptomyces albus* J1074::cos4, a transformant, which harbors a cosmid clone of the entire gene cluster of rishirilide B. *Streptomyces albus* J1074::cos4 was cultivated in TSB media (CASO bouillon 30 g·L^−1^, Carl Roth, Karlsruhe, Germany) supplemented with appropriate antibiotics and incubated in shake flasks at 28 °C for 24 h.

### 4.2. Feeding Experiments

Sodium[1-^13^C]acetate, sodium[1,2-^13^C_2_]acetate and sodium[2-^13^C]acetate were purchased from Sigma Aldrich and have 99% ^13^C atom purity, whereas [^13^c_5_,^15^n_1_]-l-valine was purchased from cortecnet (98% ^13^C, 95% ^15^N enriched). Feeding experiments with labelled sodium[1-^13^C]acetate, sodium[1,2-^13^C_2_]acetate and sodium[2-^13^C] acetate were performed in HA media (glucose 4 g·L^−1^, yeast extract 4 g·L^−1^, malt extract 10 g·L^−1^, pH 7.4), whereas experiments with [^13^c_5_,^15^n_1_]-l-valine were carried out in DNPM media (Bacto Soytone 7.5 g·L^−1^, dry yeast 5 g·L^−1^, MOPS 21 g·L^−1^, pH 6.8) both supplemented with appropriate antibiotics. The media was inoculated (1% *v*/*v*) with a 24 h old TSB culture of *Streptomyces albus* J1074::cos4. After 20 h of cultivation, determined as the starting point of rishirilide B production, the isotope labelled precursors were added aseptically into the production media. The feeding was done in 10 pulses—every six hours with a 12 h overnight break, hence 3 feedings within 24 h. The final concentration for [1-^13^C]acetate, [1,2-^13^C_2_]acetate and [2-^13^C]acetate was 6.1 mM and for feeding with [^13^c_5_,^15^n_1_]-l-valine, 2 mM. Rishirilide B was isolated after 5–6 days when production reaches its maximum.

### 4.3. Analysis of Rishirilide B by HPLC/MS

Rishirilide B production was monitored on a HPLC/MS system equipped with a XBridge C18 precolumn (3.5 μm; 20 mm × 4.6 mm) and an XBridge C18 main column (3.5 μm, 100 mm × 4.6 mm) coupled to a DAD UV detector and MS (Agilent, 1100 series, Agilent Technologies, Waldbronn, Germany). The column was run at a flow rate of 0.5 mL·min^−1^ beginning with 80% of buffer A (H_2_O + 0.5% acetic acid) and 20% of buffer B (MeCN + 0.5% acetic acid). After 6 min, buffer B was raised to 30% within 1 min followed by an 18 min linear gradient from 30% to 95%. After a delay of 3 min at 95%, the conditions returned to the starting values within 2 min followed by 5 min equilibration. Rishirilide B was detected at 254 nm.

### 4.4. Isolation and Purification of Rishirilide B

Rishirilide B was isolated from 2 L of culture broth for all feeding experiments. After adjustment to pH 4 the culture broth was centrifuged and extracted with EtOAc. The crude extract was fractionated with increasing methanol content (10% increments) by solid phase extraction (Oasis HLB 35cc (6g) LP Extraction Cartridge, Waters GmbH, Eschborn, Germany). The 70% and 80% methanol fractions containing rishirilide B were combined and further purified by semi preparative HPLC using a Zorbax SB-C18 precolumn (5 μm, 9.4 mm × 20 mm) and a Zorbax B-C18 main column (5 μm, 9.4 mm × 150 mm) coupled to a PDA detector. The column was eluted with buffer A and buffer B at a flow rate of 0.5 mL·min^−1^.

The gradients for purification of rishirilide B from acetate feeding and l-valine feeding experiments were slightly different. The starting conditions for purification of rishirilide B from acetate feeding experiments were 50% buffer A and 50% buffer B held for 4 min followed by a 7 min linear gradient to 95% buffer B, where rishirilide B was collected. With a delay of 1 min at 95% buffer B, the conditions were changed to starting conditions and maintained for 15 min.

The starting conditions for purification of rishirilide B from l-valine feeding experiment were 55% buffer A and 45% buffer B. After 3 min the concentrations were changed to 80% buffer B over a 6 min linear gradient. After 9 min, buffer B was quickly increased to 95%. With a delay of 3 min under these conditions, buffer B was lowered to starting conditions at minute 12, and the column was washed for a further 3 min.

Finally, the samples were further purified on a column (39.5 cm × 1.4 cm) packed with Sephadex^®^ LH20 (GE Healthcare GmbH, Solingen, Germany) with MeOH as solvent. Flow rate was determined by gravity. The fractions were analyzed by HPLC-MS and rishirilide B containing fractions were combined and evaporated to dryness.

### 4.5. NMR Analysis of Rishirilide B

NMR spectra were recorded using a Varian VNMR-S 600 equipped with a Nalorac 3 mm broadband probe. Spectra were recorded in 150 µL DMSO-d_6_ at 35 °C. Solvent signals were used as internal standard (DMSO-d_6_: δ_H_ = 2.50 ppm, δ_C_ = 39.5 ppm). Calculation of enrichment and specific enrichment was done according to Scott et al. [22].

## 5. Conclusions

Investigations of rishirilide B biosynthesis have been carried out from feeding experiments with different isotopic labelled acetates and l-valine. The carbon skeleton of rishirilide B is assembled from nine acetate units and one isobutyrate (derived from valine). One acetate undergoes decarboxylation to afford a methyl group. The pattern of the labelling positions from feeding experiments with labelled acetate reveals that a rearrangement of the carbon skeleton occurs during rishirilide B biosynthesis. Future studies involving gene knock out experiments and isolation of reaction intermediates will provide insight into the enzymes and mechanisms involved in this rearrangement.

## Figures and Tables

**Table 1:** ^13^C-NMR signals of rishirilide B together with specific incorporations and coupling constants after feeding with [1-^13^C]acetate (I), [2-^13^C]acetate (II), [1,2-^13^C_2_]acetate (III) and [^13^c_5_,^15^n]-l-valine (IV).

Pos.	δ_C_ (ppm)	I^(b)^ (%)	II^(a)^ (%)	III J_CC_ (Hz)	III^(c)^ J Partner	IV J_CC_ (Hz)	IV J Partner
1	197.3	−0.4	3.2	41	C-2		^(d)^
2	47.9	3.8	−0.6	41	C-1		^(d)^
3	83.0	0.8	4.4	51	C-16		^(d)^
4	76.6	4.9	−0.3	39	C-11		^(d)^
4a	140.8	−	6.3	64	C-10		^(d)^
5	152.9	4.6	−0.5	64	C-10a		^(d)^
6	109.9	−0.4	2.6	55	C-7		^(d)^
7	126.0	7.9	−0.4	55	C-6		^(d)^
8	119.6	−0.3	1.3	55	C-8a		^(d)^
8a	132.2	5.1	−0.6	55	C-8		^(d)^
9	125.3	0.4	3.8	64	C-9a		^(d)^
9a	130.3	4.2	0.4	64	C-9		^(d)^
10	119.3	6.7	−0.3	64	C-10a		^(d)^
10a	126.3	−0.8	2.0	64	C-10		^(d)^
11	35.0	−0.3	2.4	39	C-4		^(d)^
12	31.1	−0.03	−0.2	-	-	35	C-13^(c)^
13	27.9	−0.2	−0.1	-	-	35,35	C-12^(c)^, C-14^(c)^, C-15^(c)^
14	22.4	^(b)^	^(b)^	-	-	35	C-13^(c)^
15	22.6	−0.1	−0.4	-	-	35	C-13^(c)^
16	173.8	5.0	−0.1	51	C-3		^(d)^
17	10.1	−0.3	1.4	-	-		^(d)^

(**a**) Relative enrichments were normalized to peak intensity of the C-14 signal; (**b**) Reference signal; (**c**) Supported from Inadequate NMR spectrum; (**d**) Weak enrichment, pattern similar to [1,2-^13^C_2_]acetate incorporation through biosynthetic transformation of valine.

## Supplementary Materials

The following are available online at http://www.mdpi.com/2079-6382/7/1/20/s1; Table S1: NMR data of rishirilide B (600/150 MHz, DMSO-d_6_, 35 °C); Table S2: Calculation of enrichment and specific enrichment for rishirilide B from feeding experiment with [1-^13^C]acetate; Figure S1: Labelling positions of rishirilide B after feeding experiment with [1-^13^C]acetate; Figure S2: ^13^C NMR spectra (150 MHz, DMSO-d_6_, 35 °C) of rishirilide B after feeding of [1-^13^C]acetate in comparison to rishirilide B at natural abundance; Table S3: Table of NMR data of rishirilide B from feeding experiment with [1,2-^13^C_2_]acetate; Figure S3: Labelling positions of rishirilide B after feeding experiment with [1,2-^13^C_2_]acetate; Figure S4: ^13^C NMR spectra (150 MHz, DMSO-d_6_, 35 °C) of rishirilide B after feeding of [1,2-^13^C_2_]acetate in comparison to rishirilide B at natural abundance; Figure S5: Inadequate (150 MHz, DMSO-d_6_, 35 °C) of rishirilide B after feeding of [1,2-^13^C_2_]acetate; Table S4: Results of feeding experiment with l-[methyl-^13^c]methionine; Figure S6: ^13^C NMR spectra (150 MHz, DMSO-d_6_, 35 °C) of rishirilide B from feeding experiment with l-[methyl-^13^c]methionine in comparison to rishirilide B at natural abundance; Table S5: Calculation of enrichment and specific enrichment for rishirilide B from feeding experiment with [2-^13^C]acetate; Figure S7: Labelling positions of rishirilide B after feeding experiment with [2-^13^C]acetate; Figure S8: ^13^C NMR spectra (150 MHz, DMSO-d_6_, 35 °C) of rishirilide B after feeding of [2-^13^C]acetate in comparison to rishirilide B at natural abundance; Figure S9: Mass spectrum of rishirilide B from feeding experiment with [^13^c_5_, ^15^n_1_]-l-valine; Table S6: Table of NMR data rishirilide B from feeding experiment with [^13^c_5_, ^15^n_1_]-l-valine; Figure S10: Labelling positions of rishirilide B after feeding experiment with [^13^c_5_, ^15^n_1_]-l-valine; Figure S11: ^13^C NMR spectra (150 MHz, DMSO-d_6_, 35 °C) of rishirilide B from feeding experiment with [^13^c_5_, ^15^n_1_]-l-valine in comparison to rishirilide B at natural abundance; Figure S12: Inadequate (150 MHz, DMSO-d_6_, 35 °C) of rishirilide B from feeding experiment with [^13^c_5_, ^15^n_1_]-l-valine; Figure S13: Expansion of Inadequate (150 MHz, DMSO-d_6_, 35 °C) of rishirilide B from feeding experiment with [^13^c_5_, ^15^n_1_]-l-valine.
