# Supplementary material for: Biosynthesis of Rishirilide B
Source: Antibiotics (Basel). 2018 Mar 7;7(1):20. doi: 10.3390/antibiotics7010020 (PMC5872131; doi:10.3390/antibiotics7010020)

Supporting Informations

Biosynthesis of Rishirilide B

Philipp Schwarzer [1,#], Juli Wunsch-Palasis [1,#], Andreas Bechthold [1,*] and Thomas Paululat [2,*]

1 P. Schwarzer, Dr. J. Wunsch-Palasis, Prof. Dr. A. Bechthold, Pharmaceutical Biology and Biotechnology, University of Freiburg, Stefan-Meier-Str. 19, 79104 Freiburg i. Br. (Germany); andreas.bechthold@pharmazie.uni-freiburg.de

2 Dr. T. Paululat, Organic Chemistry, University of Siegen, Adolf-Reichwein-Str. 2, 57068 Siegen (Germany); thomas.paululat@uni-siegen.de

# P. Schwarzer and Dr. J. Wunsch-Palasis contributed equally to this work

* Correspondence: andreas.bechthold@pharmazie.uni-freiburg.de and thomas.paululat@uni-siegen.de

Tables and Figures:

Table S1:	NMR data of rishirilide B (600/150 MHz, DMSO-d_6, 35 °C)
Table S2:	Calculation of enrichment and specific enrichment for rishirilide B from feeding experiment with [1-^{13}C]acetate
Figure S1:	Labelling positions of rishirilide B after feeding experiment with [1-^{13}C]acetate
Figure S2:	^{13}C NMR spectra (150 MHz, DMSO-d_6, 35 °C) of rishirilide B after feeding of [1-^{13}C]acetate in comparison to rishirilide B at natural abundance
Table S3:	Table of NMR data of rishirilide B from feeding experiment with [1,2-$^{13}C_2$]acetate
Figure S3:	Labelling positions of rishirilide B after feeding experiment with [1,2-$^{13}C_2$]acetate
Figure S4:	^{13}C NMR spectra (150 MHz, DMSO-d_6, 35 °C) of rishirilide B after feeding of [1,2-$^{13}C_2$]acetate in comparison to rishirilide B at natural abundance
Figure S5:	Inadequate (150 MHz, DMSO-d_6, 35 °C) of rishirilide B after feeding of [1,2-$^{13}C_2$]acetate
Table S4:	Results of feeding experiment with L-[methyl-^{13}C]methionine
Figure S6:	^{13}C NMR spectra (150 MHz, DMSO-d_6, 35 °C) of rishirilide B from feeding experiment with L-[methyl-^{13}C]methionine in comparison to rishirilide B at natural abundance
Table S5:	Calculation of enrichment and specific enrichment for rishirilide B from feeding experiment with [2-^{13}C]acetate
Figure S7:	Labelling positions of rishirilide B after feeding experiment with [2-^{13}C]acetate
Figure S8:	^{13}C NMR spectra (150 MHz, DMSO-d_6, 35 °C) of rishirilide B after feeding of [2-^{13}C]acetate in comparison to rishirilide B at natural abundance
Figure S9:	Mass spectrum of rishirilide B from feeding experiment with [$^{13}C_5$, $^{15}N_1$]-L-valine
Table S6:	Table of NMR data rishirilide B from feeding experiment with [$^{13}C_5$, $^{15}N_1$]-L-valine
Figure S10:	Labelling positions of rishirilide B after feeding experiment with [$^{13}C_5$, $^{15}N_1$]-L-valine
Figure S11:	^{13}C NMR spectra (150 MHz, DMSO-d_6, 35 °C) of rishirilide B from feeding experiment with [$^{13}C_5$, $^{15}N_1$]-L-valine in comparison to rishirilide B at natural abundance
Figure S12:	Inadequate (150 MHz, DMSO-d_6, 35 °C) of rishirilide B from feeding experiment with [$^{13}C_5$, $^{15}N_1$]-L-valine
Figure S13:	Expansion of Inadequate (150 MHz, DMSO-d6, 35 °C) of rishirilide B from feeding experiment with [$^{13}C_5$, $^{15}N_1$]-L-valine.

Table S1: NMR data of rishirilide B (600/150MHz, DMSO-d_6, 35 °C)

Pos.	δ_C [ppm]	δ_H (J Hz) [ppm]	COSY[a]	HMBC and bsgHMBC(115-135ppm)
1	197.1			9-H, 2-H, 17-H_3
2	47.9	2.99 q (6.8)	17-H_3	17-H_3
3	83.6			2-H, 17-H_3
4	76.9			10-H, (11-H_a)
4a	140.0			9-H, (11-H_a)
5	153.0			6-H, 7-H, 8-H, 10-H
6	109.9	6.93 d (7.6)	7-H	8-H
7	126.3	7.28 dd (8.3, 7.6)	6-H, 8-H	6-H, 8-H
8	119.7	7.46 d (8.3)	7-H	6-H, 9-H
8a	132.3			7-H, 8-H, 10-H
9	125.7	8.29 s	(8-H)	8-H
9a	129.9			10-H
10	119.6	8.28 s		6-H, 9-H
10a	126.1			6-H, 8-H, 9-H
11	35.0	11-H_a: 2.23 dt (13.1, 3.9)	11-H_b, 12-H_a, 12-H_b	12-H_b
		11-H_b: 1.61 ddd (13.3, 12.8, 4.7)	11-H_a, 12-H_a, 12-H_b	
12	31.1	12-H_a: 1.38 m	11-H_a, 11-H_b	11-H_b, 13-H, 14-H_3, 15-H_3
		12-H_b: 0.78 m	11-H_a, 11-H_b, 13-H	
13	27.8	1.30 m	12-H_b, 14-H_3, 15-H_3	12-H_a, 14-H_3, 15-H_3
14	22.4	0.66 d (6.5)	13-H	15-H_3
15	22.6	0.77 d (6.5)	13-H	12-H_a, 13-H, 14-H_3
16	174.0			2-H
17	10.1	1.19 d (6.8)	2-H	2-H
OH		10.2 s br		

[a]weak signals in brackets

Table S2: Calculation of enrichment and specific enrichment for rishirilide B from feeding experiment with [1-^{13}C]acetate

Pos.	δ_C [ppm]	Intensity reference signal	Intensity from feeding experiment	Normalized intensities from feeding experiment (factor: 1.318)	Enrichment (specific enrichment) [%]
1	197.3	11.0	5.6	7.4	-0.36
2	47.9	29.5	99.1	131.4	3.8 (3.8)
3	83.0	5.7	7.7	10.1	0.8
4	76.6	15.7	64.6	85.1	4.9 (4.9)
4a	140.8	4.1	-	Missing signal	
5	152.9	24.5	97.1	128.0	4.6 (4.6)
6	109.6	26.9	12.1	15.9	-0.4
7	126.0	19.8	123.4	162.6	7.9 (8.0)
8	119.6	26.9	15.4	20.3	-0.3
8a	132.2	21.4	91.2	120.2	5.1 (5.2)
9	125.3	17.5	8.8	11.6	0.4
9a	130.3	7.7	28.4	37.4	4.2 (4.2)
10	119.3	25.0	134.0	176.6	6.7 (6.8)
10a	126.3	19.5	4.4	5.8	-0.8
11	35.0	29.1	16.6	21.9	-0.3
12	31.1	31.1	22.9	30.2	-0.03
13	27.9	40.2	25.0	33.0	-0.2
14 [c]	22.4	51.8	39.3	51.8	[a]
15	22.6	60.3	41.5	54.7	-0.1
16	173.8	11.5	48.0	63.3	5.0 (5.1)
17	10.1	41.0	23.1	30.4	-0.3

[a]reference signal for normalization

Figure S1: Labelling positions of rishirilide B after feeding experiment with [1-^{13}C]acetate

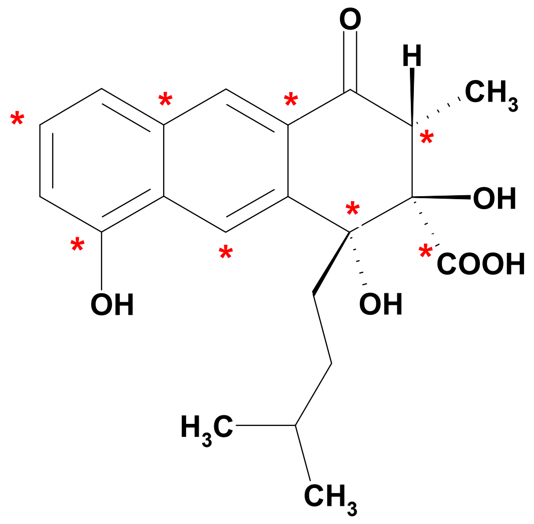

Figure S2: ^{13}C NMR spectra (150MHz, DMSO-d_6, 35 °C) of rishirilide B after feeding of [1-^{13}C]acetate (red) in comparison to rishirilide B at natural abundance (black)

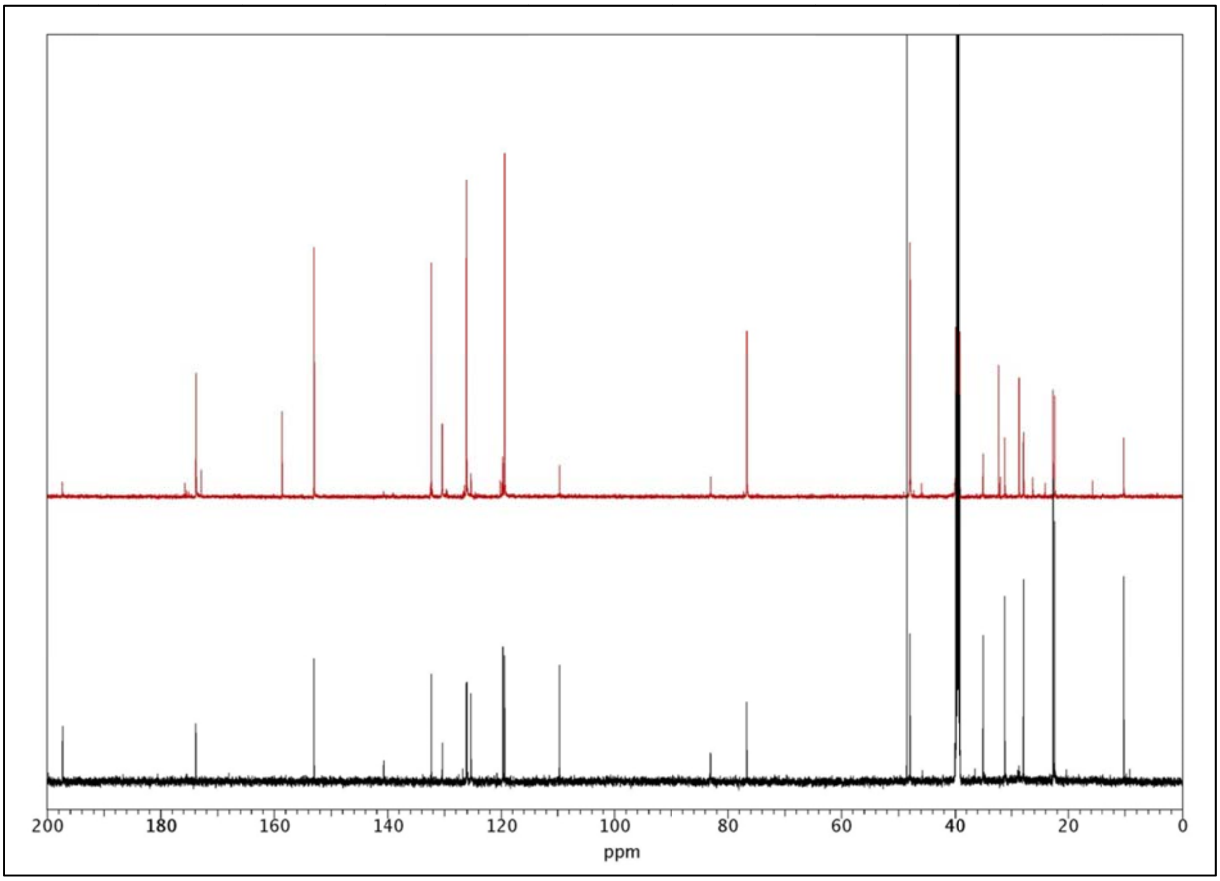

Table S3: Table of NMR data of rishirilide B from feeding experiment with [1,2-$^{13}C_2$]acetate

Pos.	δ_C [ppm]	J_{CC} [Hz]	Inadequate
1	197.3	41	C-2
2	47.9	41	C-1
3	83.0	51	C16
4	76.6	39	C-11
4a	140.8	64	C-10
5	152.9	64	C-10a
6	109.9	55	C-7
7	126.0	55	C-6
8	119.6	55	C-8a
8a	132.2	55	C-8
9	125.3	64	C-9a
9a	130.3	64	C-9
10	119.3	64	C-10a
10a	126.3	64	C-10
11	35.0	39	C-4
12	31.1	-	-
13	27.9	-	-
14	22.4	-	-
15	22.6	-	-
16	173.8	51	C-3
17	10.1	-	-

Figure S3: Labelling positions of rishirilide B after feeding experiment with [1,2-$^{13}C_2$]acetate

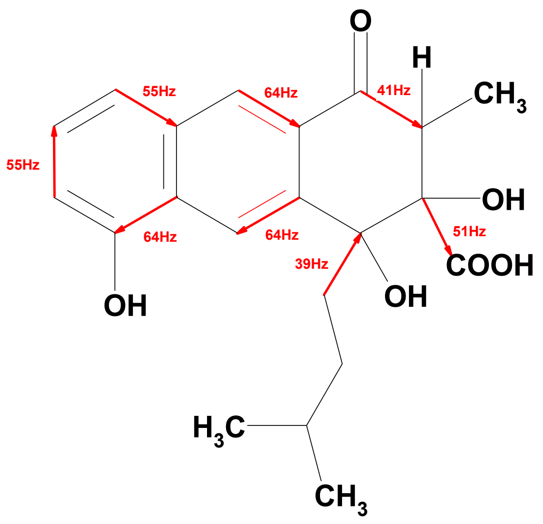

Figure S4: ^{13}C NMR spectra (150MHz, DMSO-d_6, 35 °C) of rishirilide B after feeding of [1,2-$^{13}C_2$]acetate (red) in comparison to rishirilide B at natural abundance (black)

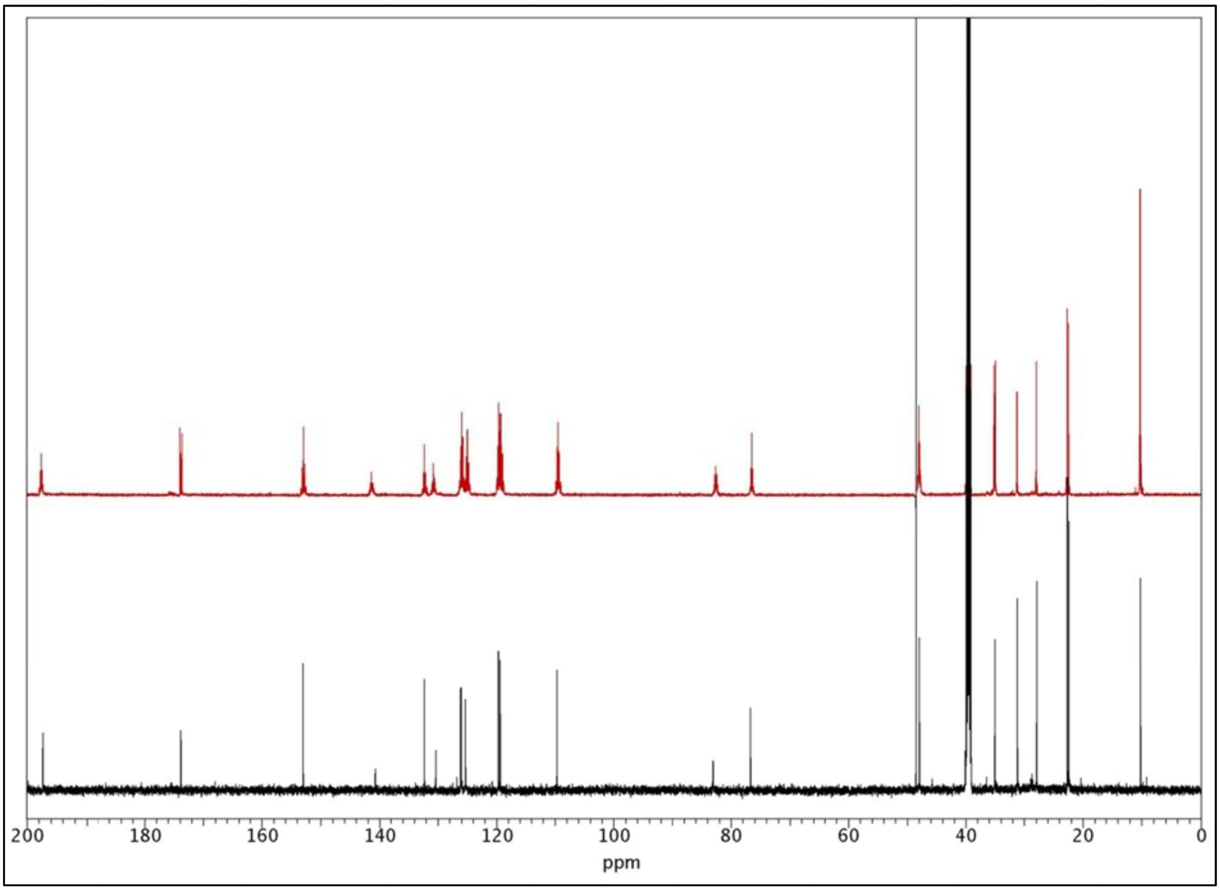

Figure S5: Inadequate (150MHz, DMSO-d_6, 35 °C) of rishirilide B after feeding of [1,2-$^{13}C_2$]acetate

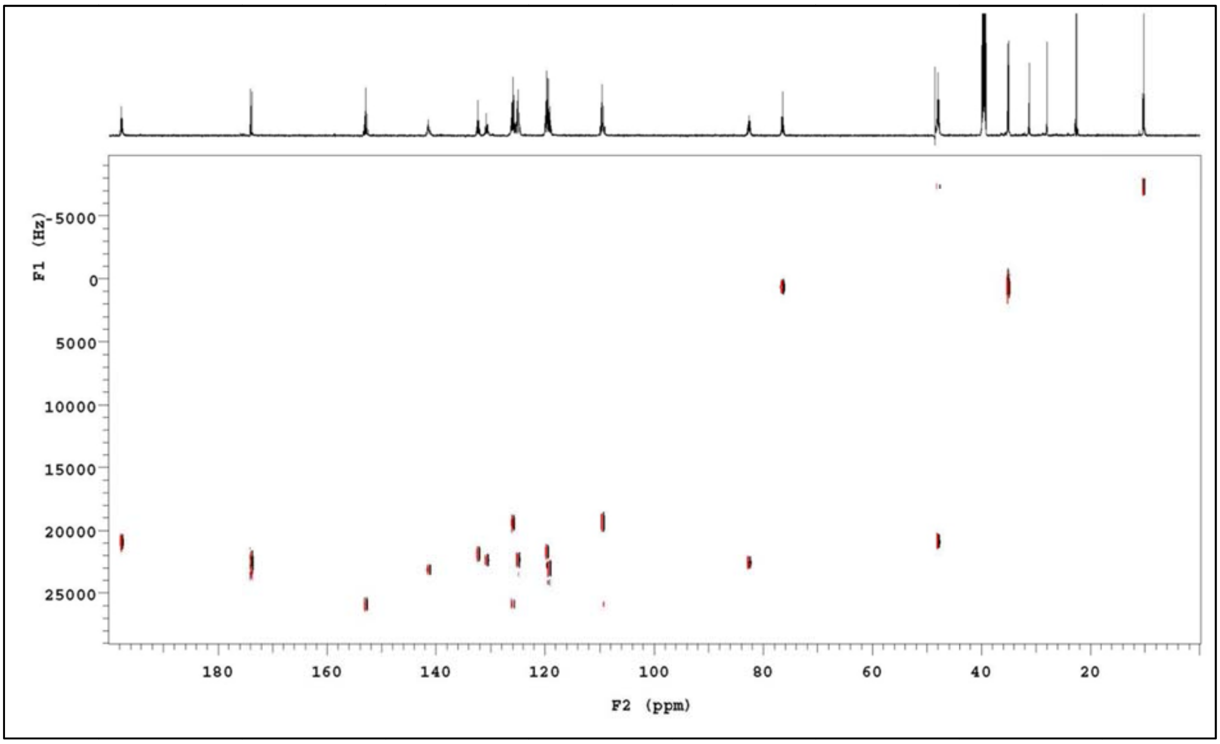

Table S4: Results of feeding experiment with L-[methyl-^{13}C]methionine

Pos.	δ_C [ppm]	Intensity reference signal	Intensity from feeding experiment	Normalized intensities from feeding experiment (factor: 0.985)	Enrichment (specific enrichment) [%]
1	197.3	28.1	31.0	27.5	-0.05
2	47.9	75.5	68.4	60.3	-0.22
3	83.0	14.6	17.9	15.8	0.09
4	76.6	40.4	49.7	43.8	0.09
4a	140.8	10.6	21.7	18.7	0.84
5	152.9	62.7	69.6	61.4	-0.02
6	109.6	59.4	81.0	71.4	0.22
7	126.0	50.9	64.1	56.5	0.12
8	119.6	68.9	88.4	78.0	0.14
8a	132.2	54.7	67.2	59.3	0.09
9	125.3	44.8	63.7	56.4	0.29
9a	130.3	19.7	34.3	30.3	0.59
10	119.3	64.2	71.5	63.1	0.02
10a	126.3	49.9	68.8	60.7	0.23
11	35.0	74.5	84.5	74.5	[a]
12	31.1	94.5	111.6	98.4	0.05
13	27.9	103.2	142.0	125.8	0.24
14	22.4	132.9	171.1	150.9	0.15
15	22.6	154.5	189.5	167.1	0.09
16	173.8	29.4	27.2	24.0	-0.20
17	10.1	105.0	105.3	92.9	-0.13

[a]Reference Signal for normalization and calculation of enrichment

No labelled position could be observed.

Figure S6: ^{13}C NMR spectra (150MHz, DMSO-d_6, 35 °C) of rishirilide B from feeding experiment with L-[methyl-^{13}C]methionine (red) in comparison to rishirilide B at natural abundance (black)

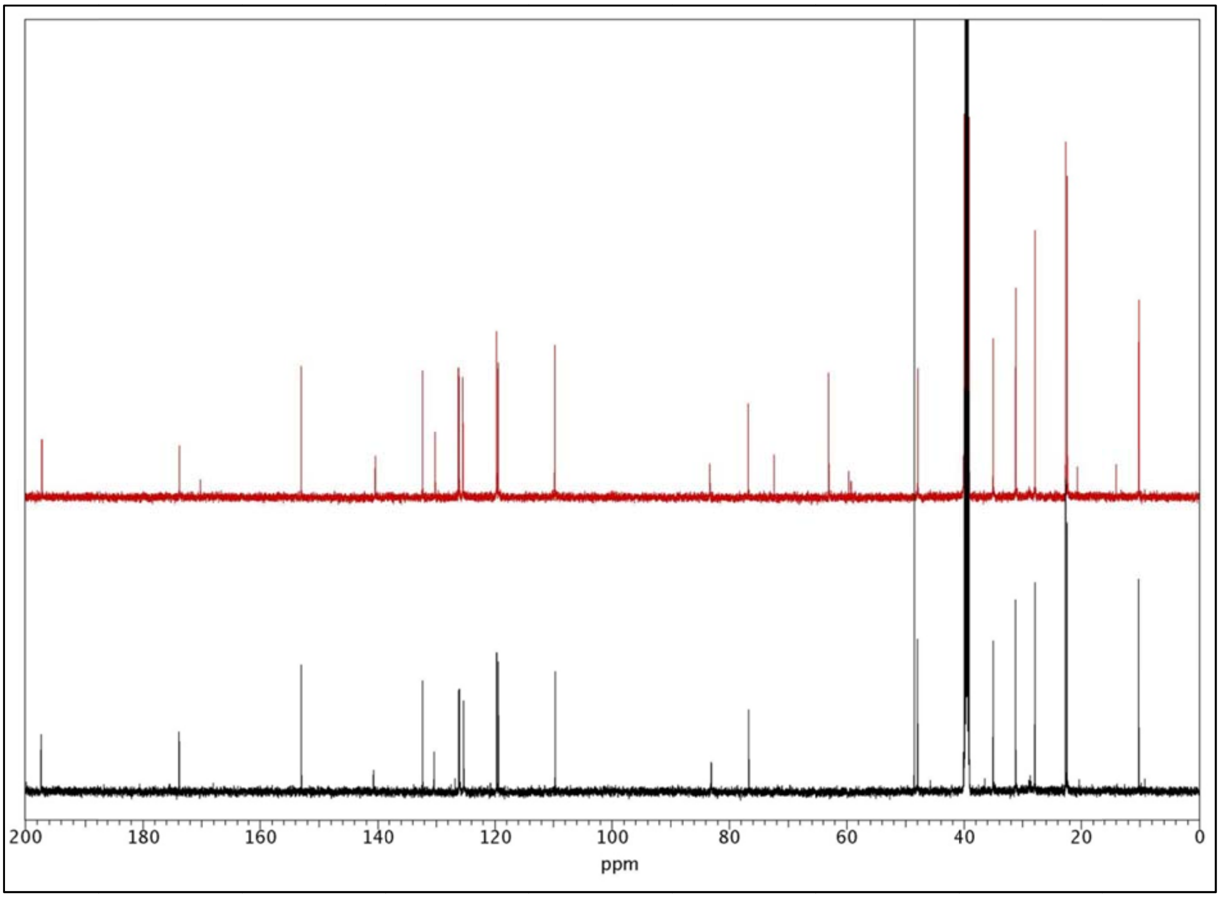

No enrichment visible

Table S5: Calculation of enrichment and specific enrichment for rishirilide B from feeding experiment with [2-^{13}C]acetate

Pos.	δ_C [ppm]	Intensity reference signal	Intensity from feeding experiment	Normalized intensities from feeding experiment (factor: 0.985)	Enrichment (specific enrichment) [%]
1	197.3	11.0	43.9	43.2	3.2 (3.3)
2	47.9	29.5	14.4	14.2	-0.6
3	83.0	5.7	28.8	28.4	4.4 (4.5)
4	76.6	15.7	11.4	11.2	-0.3
4a	140.8	4.1	27.9	27.5	6.3 (6.4)
5	152.9	24.5	13.7	13.5	-0.5
6	109.6	23.2	79.7	78.5	2.6 (2.7)
7	126.0	19.5	12.6	12.4	-0.4
8	119.6	26.9	60.0	59.1	1.3 (1.3)
8a	132.2	21.4	10.8	10.6	-0.6
9	125.3	17.5	79.2	78.0	3.8 (3.8)
9a	130.3	7.7	10.8	10.6	0.4
10	119.3	25.0	18.5	18.2	-0.3
10a	126.3	19.8	56.6	55.8	2.0 (2.0)
11	35.0	29.1	94.3	92.9	2.4 (2.4)
12	31.1	31.1	25.4	25.0	-0.2
13	27.9	40.2	37.9	37.3	-0.1
14 [a]	22.4	51.8	52.6	51.8	[a]
15	22.6	60.3	38.9	38.3	-0.4
16	173.8	11.5	10.7	10.5	-0.1
17	10.1	41.0	93.5	92.1	1.4 (1.4)

[a]Reference Signal for normalization and calculation of enrichment

Figure S7: Labelling positions of rishirilide B after feeding experiment with [2-^{13}C]acetate

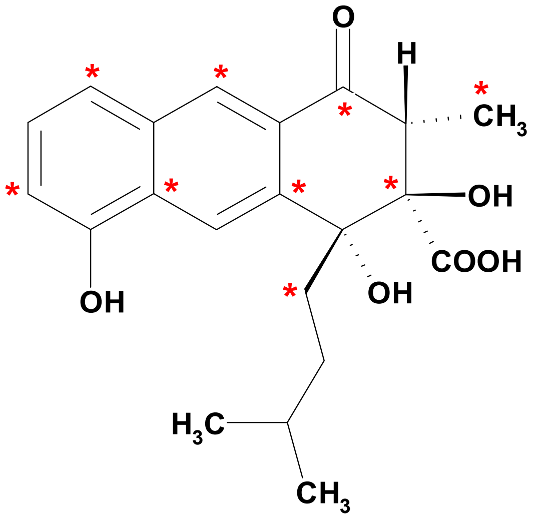

Figure S8: ^{13}C NMR spectra (150MHz, DMSO-d_6, 35 °C) of rishirilide B after feeding of [2-^{13}C]acetate (red) in comparison to rishirilide B at natural abundance (black)

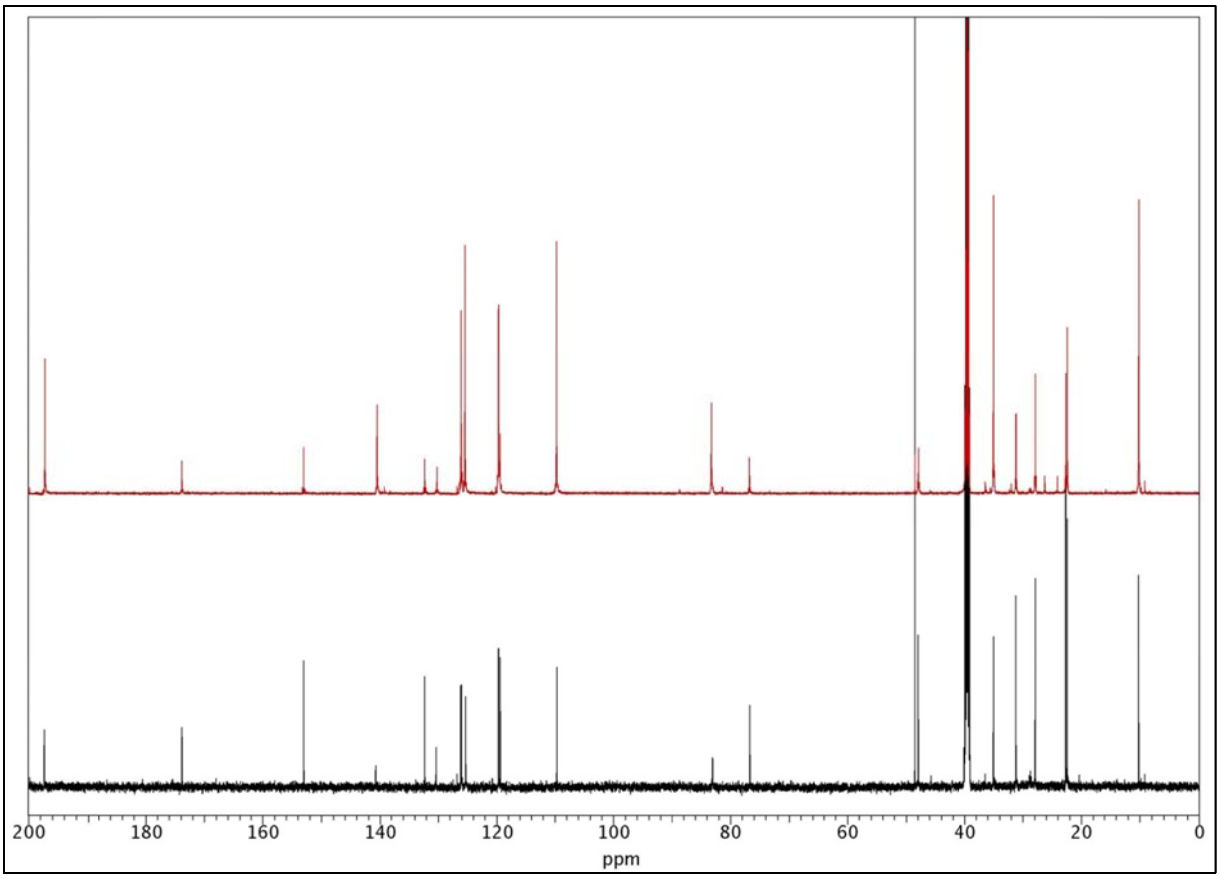

Figure S9: Mass spectrum of rishirilide B from feeding experiment with [$^{13}C_5$, $^{15}N_1$]-L-valine

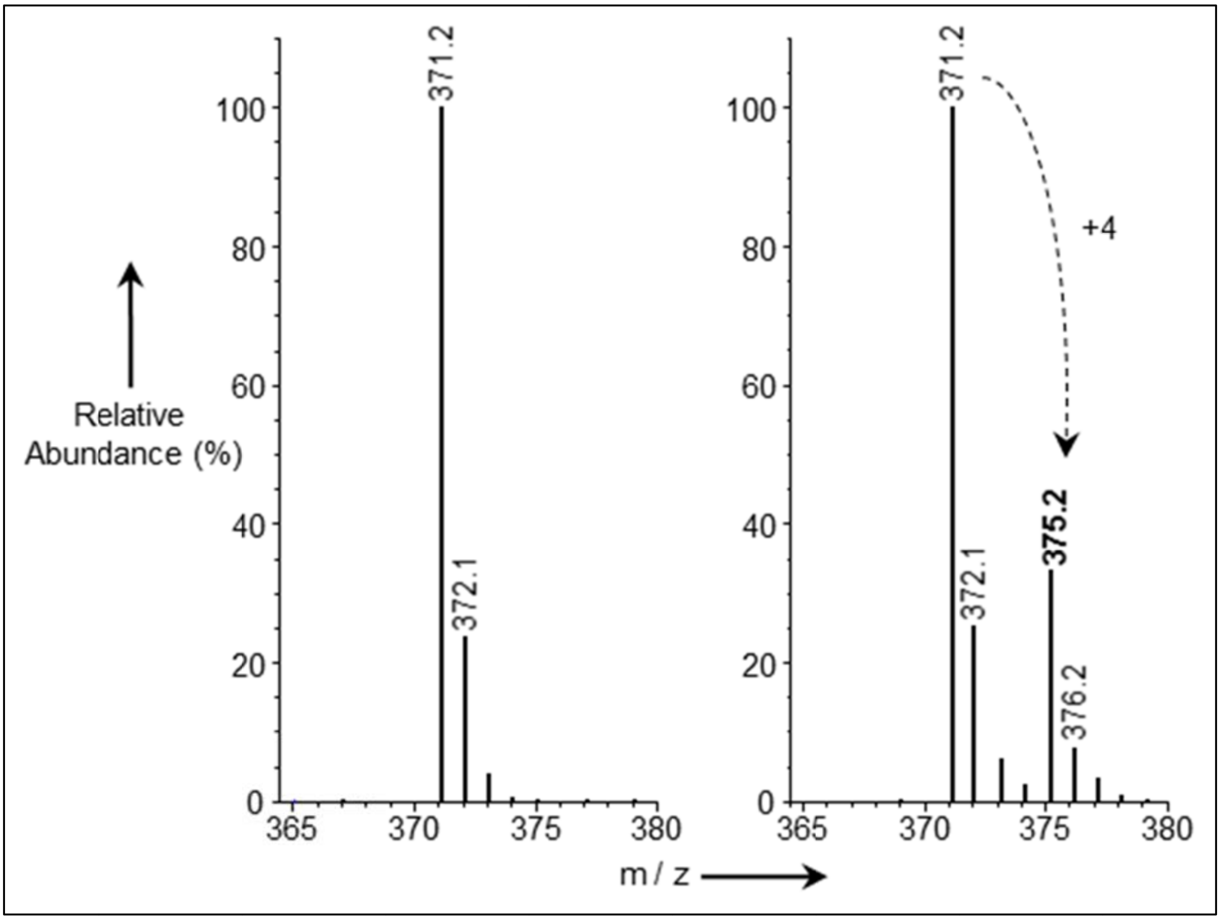

Table S6: Table of NMR data of rishirilide B from feeding experiment with [$^{13}C_5$, $^{15}N_1$]-L-valine

Pos.	δ_C [ppm]	Enrichment[a] (strong or weak)	J_{CC}[a] [Hz]	Multiplicity[a]	Inadequate[b]
1	197.3	Weak	40	d	-
2	47.9	Weak	40	d	-
3	83.0	Weak	55	d	-
4	76.6	Weak	37	d	-
4a	140.8	Weak	63	d	-
5	152.9	Weak	64	d	-
6	109.9	Weak	56	d	-
7	126.0	Weak	56	d	-
8	119.6	Weak	55	d	-
8a	132.2	Weak	55	d	-
9	125.3	Weak	62	d	-
9a	130.3	Weak	62	d	-
10	119.3	Weak	63	d	-
10a	126.3	Weak	64	d	-
11	35.0	Weak	37	d	-
12	31.1	Strong	35	d	C-13
13	27.9	Strong	35, 35	dt	C-12, C-14, C-15
14	22.4	Strong	35	d	C-13
15	22.6	Strong	35	d	C-13
16	173.8	Weak	51	d	-
17	10.1	No	-	s	-

[a] from ^{13}C NMR spectrum
[b] from INADEQUATE NMR spectrum

Figure S10: Labelling positions of rishirilide B after feeding experiment with [$^{13}C_5$, $^{15}N_1$]-L-valine

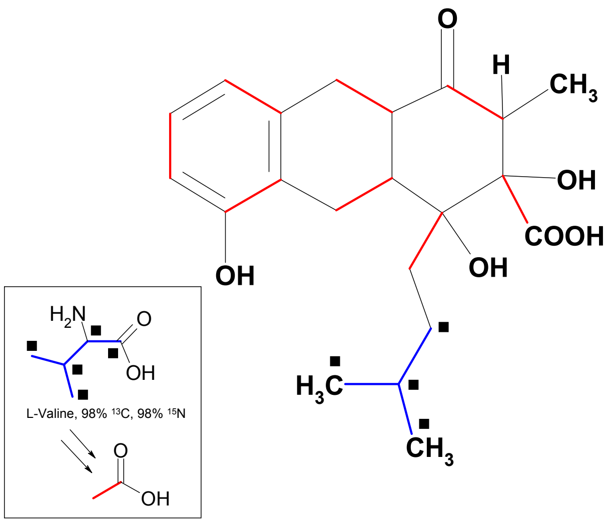

Figure S11: ^{13}C NMR spectra (150MHz, DMSO-d$_6$, 35 °C) of rishirilide B from feeding experiment with [$^{13}C_5$, $^{15}N_1$]-L-valine (red) in comparison to rishirilide B at natural abundance (black)

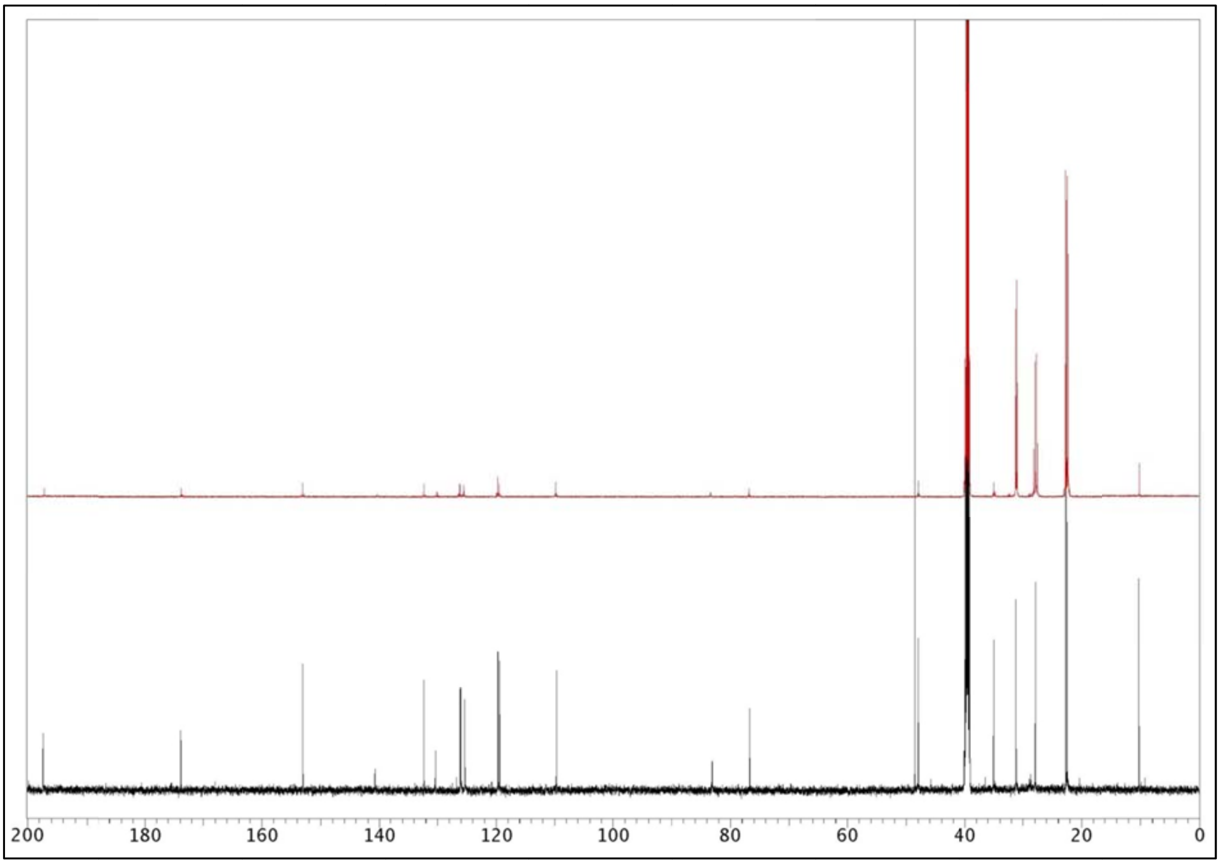

Figure S12: Inadequate (150MHz, DMSO-d$_6$, 35 °C) of rishirilide B from feeding experiment with [$^{13}C_5$, $^{15}N_1$]-L-valine

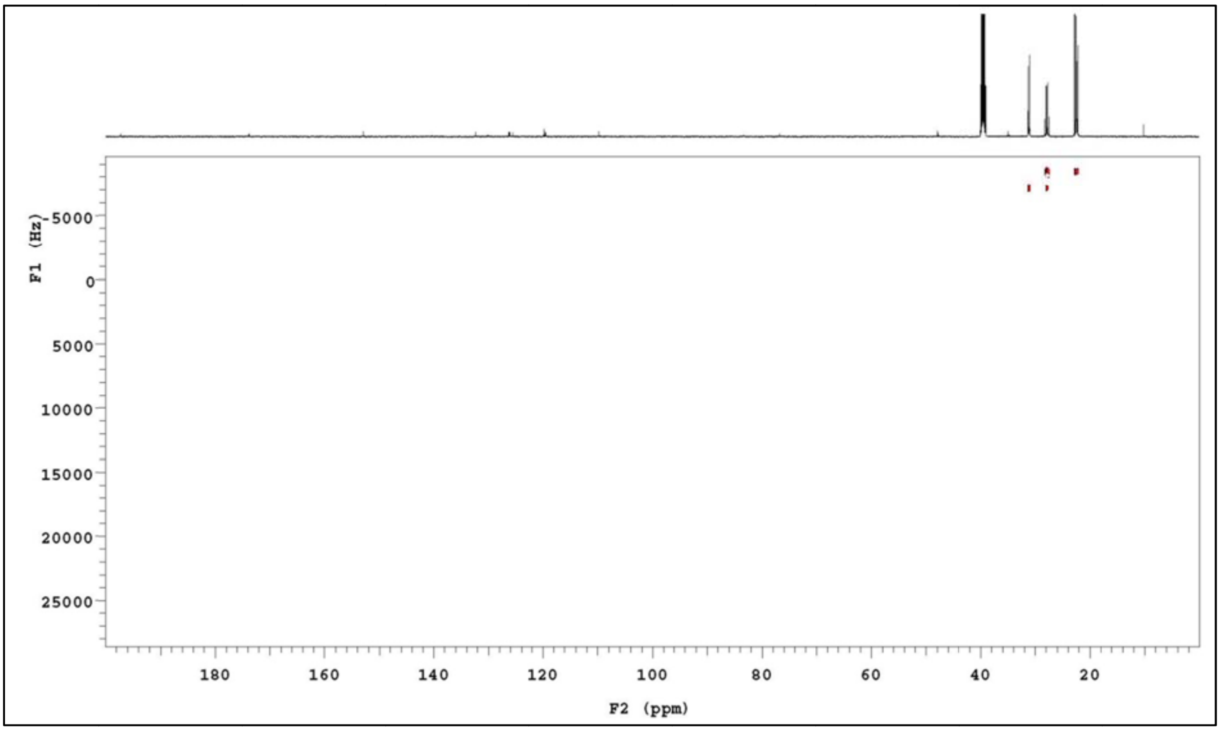

Figure S13: Expansion of Inadequate (150MHz, DMSO-d_6, 35 °C) of rishirilide B from feeding experiment with [$^{13}C_5$, $^{15}N_1$]-L-valine

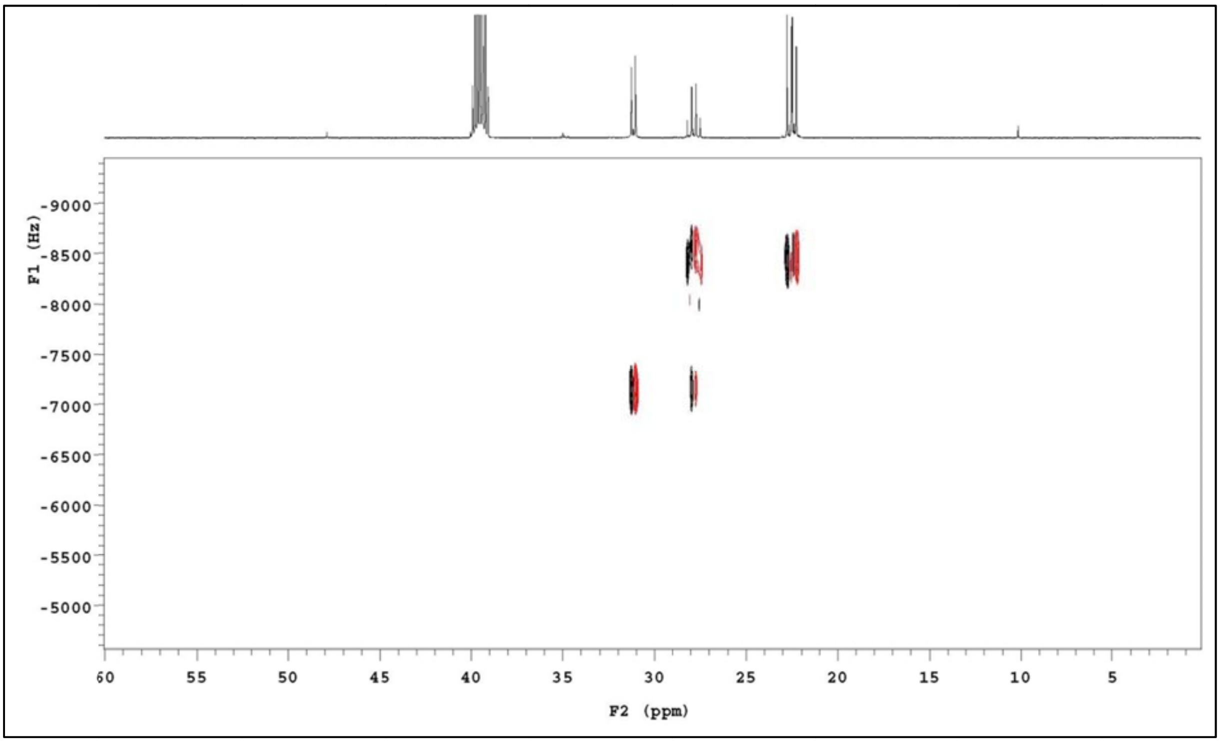

Supplement: Supplementary file 1 [file antibiotics-07-00020-s001.pdf]
